# A methylation‐related lncRNA‐based prediction model in lung adenocarcinomas

**DOI:** 10.1111/crj.13753

**Published:** 2024-08-26

**Authors:** Kun Yang, Hao Liu, Jun Hai Li

**Affiliations:** ^1^ Thoracic Surgery The Thirteenth People's Hospital of Chongqing City Chongqing City China; ^2^ Thoracic Surgery Nuclear Industry 215 Hospital Xianyang City Shaanxi Province China; ^3^ Department of Clinical Medicine Shaanxi University of Chinese Medicine Xianyang City China

**Keywords:** drug therapy, immune infiltration, lncRNAs, lung adenocarcinoma, methylation

## Abstract

**Background:**

The collaboration between methylation and the lung adenocarcinoma (LUAD) occurrence and development is closes. Long noncoding RNA (lncRNA), as a regulatory factor of various biological functions, can be used for cancer diagnosis. Our study aimed to construct a robust methylation‐related lncRNA signature of LUAD.

**Methods:**

In the Cancer Genome Atlas (TCGA) dataset, we download the RNA expression data and clinical information of LUAD cases. To develop the best prognostic signature based on methylation‐related lncRNAs, Cox regression analyses were utilized. Using Kaplan–Meier analysis, overall survival rates were compared between risk category included both low‐ and high‐risk patients. To categorize genes according to their functional significance, GSEA (Subramanian et al, 2005) was used. Single‐sample gene set enrichment analysis (ssGSEA) was used to further reveal the potential molecular mechanism of the methylation‐related lncRNA prognostic model in immune infiltration. Using TRLnc (http://www.licpathway.net/TRlnc) and lncRNASNP to analyse the SNP sites and TRLnc of these 18 lncRNAs. LncSEA website was used to analyse 18 lncRNA in the process of tumour development and development. Go was used to analyse the enriched pathways enriched by TFs (transcription factors), Cerna networks, and proteins bound to each other of these 18 lncRNAs. The ‘prophetic’ package was used to analyse the value of this prognostic model in guiding personalized immunotherapy.

**Results:**

In this study, we identified 18 methylation‐related lncRNAs (AP002761.1, AL118558.3, CH17‐340M24.3, AL353150.1, AC004687.1, LINC00996, AF186192.1, HSPC324, AC087752.3, FAM30A, AC106047.1, AC026355.1, ABALON, LINC01843, AL606489.1, NKILA, AP001453.2, GSEC) to establish a methylation‐related lncRNA signature that can detect patients prognosis in LUAD. The enriched pathways enriched by proteins interacting with 18 lncRNAs are mainly EMT, hypoxia, stemness and proliferation, among which LINC00996 and AF186192.1 are regulated by multiple tumour associated transcription factors, such as TP53 and TP63, and fam30a and mRNA form a Cerna network. There are 2319 SNP loci in LINC00996, 36 of which are risk SNP loci and 205 SNP loci in af186192.1; AF186192.1 affects 95 conserved miRNAs and 123 non‐conserved miRNAs, promotes the binding of 149 pairs of miRNAs: lncRNAs and inhibits the binding of 95 pairs of miRNAs: lncRNAs. The ROC curve demonstrated that the established methylation‐related lncRNA signature was more effective in predicting the prognosis of patients in LUAD than the clinicopathological parameters. Our research has confirmed that patients in the high‐risk group which was separated by the risk score model based on methylation‐related lncRNA had shorter OS. According to GSEA, the high‐risk group had a predominantly tumour‐ and immune‐related pathway enrichment. A significant association was shown by ssGSEA between predictive signature and immune status in LUAD patients. In addition, principal component analysis (PCA) demonstrated the prognostic and predictive value of our signature. The correlation between the predictive signature of methylation‐related lncRNA and IC50 of conventional chemotherapy drugs can provide personalized chemotherapy regimens for LUAD patients. Methylation‐related lncRNA signature can effectively predict DFS of patients in LUAD.

## Graphical abstract

Our study develops a prognostic signature for lung adenocarcinoma based on methylation‐related lncRNAs, using Cox regression analyses and Kaplan–Meier survival analysis. Functional significance of categorized genes was assessed with GSEA, while ssGSEA was used to reveal the potential molecular mechanism of the prognostic model in immune infiltration. The prognostic model was found to have potential value in guiding personalized immunotherapy.

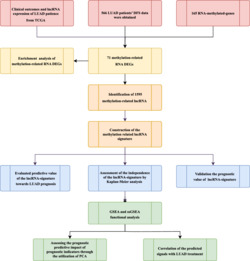

## 1 INTRODUCTION

Adenocarcinoma is China's leading cause of death. According to some statistics, globally, new cancer cases reached 19 292 789 in 2020.^1^ Approximately 3 210 000 people in China will die from cancer. Lung adenocarcinoma (LUAD) incidence and mortality have been growing very fast in China over the past 35 years. The poor prognosis and acquired drug resistance phenotype have become a difficult problem in the treatment of LUAD. Therefore, LUAD predictive markers are important for monitoring prognosis and identifying new therapeutic targets.

As a crucial regulator in epigenetics, IncRNAs affect numerous biological processes. It has been demonstrated that methylation of IncRNAs is crucial for the development of cancer. Accordingly, to explore further the role of RNA methylation regulator expression and clinical characteristics of LUAD, our research has analysed methylation modification of lncRNA by bioinformatics. The pathways enriched by methylation related lncRNA TFs and ceRNA networks and proteins bound to each other were analysed, and the TRlnc and lncRNASNP of these lncRNAs were also analysed. And we also screened key molecules to frame the prognostic model for patients with LUAD. Our results of this study provide the new mode signatures, encouraging predictive capacity on the prognosis of LUAD, and the further study basis to elucidate the mechanism research for methylation modification of lncRNAs inducing LUAD.

## 2 MATERIALS AND METHODS

### 2.1 Patient and dataset collection

We acquire raw LUAD and normal control RNA sequencing and clinical data fetched from The Cancer Genome Atlas (TCGA) (*n* = 566).^2^ Clinical outcomes and lncRNA expression values were gathered for 594 patients. According to the cBioPortal database (https://www.cbioportal.org/), 566 LUAD patients' disease‐free survival (DFS) data were obtained. The 165 methylated genes have been analysed in our study and are selected from a literature review. Six genes were methylated in m1A, and eight m5C methylated genes, 35 m6A methylated genes. There 118 m7G methylated genes were selected from gene cards (https://www.genecards.org).

### 2.2 Identification of differentially expressed methylation‐related‐lncRNA genes

The data on RNA‐methylation‐related gene expression levels were acquired from TCGA‐LUAD for the analysis. R software was used to carry out the analysis. Transcripts with log2FC| > 1 and FDR < 0.05 are selected to be differentially expressed genes (DEGs).

### 2.3 Function enrichment analysis of DEGs

We performed Gene ontology (GO) and Kyoto Encyclopedia of Genes and Genomes (KEGG) pathway analysis, to explore the biological function of DEGs and the biological pathway enriched by the DEGs.^3^

### 2.4 Development of predictive signature mediated by the methylation‐related lncRNA

The ‘limma’ software package was performed to analyse the correlation between methylation‐related genes and IncRNAs obtained from the TCGA database. Under screening standard that |*R* ^2^| > 0.3 and *P* < 0.001, 1595 methylation‐related lncRNA was gained. Following univariate COX regression analysis was used to analyse the correlation between the survival data of patients in LUAD and 1595 methylation‐related lncRNAs to screen out the lncRNAs with prognostic value. Multivariate COX regression analysis identified 18 methylation‐related lncRNAs which were used to construct the predictive signature of LUAD. In addition to calculating methylation‐related risk scores for patients in LUAD using the following formula.

The validation efficiency of the predictive signature of LUAD was evaluated according to the area under the receiver operating characteristic (ROC) curve we drew. Univariate and multivariate Cox regression analyses were performed to evaluate the independent prognostic value of the Risk model. Cluster heatmaps were further applied to evaluate the distribution relationship between clinicopathological indicators and the predictive signal was analysed to test the accuracy of the prediction model based on methylated lncRNA.

### 2.5 Analyse 18 lncRNA in the process of tumour development and development

Download ‘term set’ from the LncSEA website and use the ‘Transcription_Factor.gmt’ and ‘ceRNA.gmt’ files to analyse the TFs of these 18 lncRNAs and the ceRNA network.^4^ The file used by TF network is ‘Transcription_Factor.gmt’, the file used by ceRNA network is ‘ceRNA.gmt’ and the ‘RNA_Protein_Interaction.gmt’ file in ‘term set’ and the go package of R language are used for go enrichment analysis.

### 2.6 Analysis of the apparent regulation of methylation‐related lncRNA DEGs

We used two websites, TRlnc and lncRNASNP, to analyse the SNP sites of lncRNAs, and a total of two of 18 lncRNAs were retrieved from TRlnc: AF186192.1 and LINC00996. Among the 18 lncrnas, only LncRNASNP was retrieved: AF186192.1.

### 2.7 Construction of nomogram

For patients with LUAD, the risk score based on the methylation correlation model, age, sex, stage and N stage was incorporated into the analysis. After the analysis, we constructed a nomogram that predicts LUAD patients 1, 3 and 5 years OS after diagnosis. The calibration curve was used to describe the accuracy of predicted OS rates.^3^

### 2.8 Evaluate the independent predictive ability of lncRNA‐signature

To prove the prognostic ability of methylated‐related‐lnRNA signature independent of clinicopathological parameters, we divided LUAD patients into different subgroups according to age, gender, TNM stage and stage. Kaplan–Meier survival analysis of the subgroups. In addition, internal validation was performed to verify the prognostic value of lncRNA‐signature.

### 2.9 Functional enrichment analysis uncovered the association between methylation‐related lncRNA signature and immune condition

The patients with LUAD obtained from the TCGA database were divided into high‐risk and low‐risk groups according to the risk scores based on methylation‐related lncRNA signature. GSEA was used to analyse the functional enrichment differences of immune pathways in patients with different risk groups.ssGSEA programme was performed by the ‘GSVA’ R package to quantify the differences in 16 immune cell infiltration levels and 13 immune pathway activities between patients in different groups. Expression levels of Immune checkpoint genes in different risk groups were analysed using the Wilcoxon test.

### 2.10 Evaluation of methylation‐related lncRNA signature in clinical treatment

The ‘prophetic’ software package was used to analyse the commonly used immunotherapy drugs in the clinical treatment of LUAD. Wilcoxon signed‐rank test was performed to compare the half maximal inhibitory concentration (IC50) values between the high‐risk group and the low‐risk group to evaluate the effectiveness of the predictive signature in predicting the prognosis of LUAD.

### 2.11 Statistical analysis

Throughout this article, R software is carried out for all analyses. Using the Wilcoxon test, the difference between tumour and normal tissues was analysed. Risk prognostic models were constructed using the Cox regression algorithm. Multivariate Cox regression algorithms were performed to show the independent prognostic value of the Risk model. The Chi‐square test was applied to draw a heatmap, which reveal the distribution and expression of 18 methylation‐related lncRNAs and clinicopathological factors in different risk groups.^5^ Using Kaplan–Meier analyses estimates the OS time of patients in different risk groups. R package ‘survivalROC’ was used to generate the receiver operating characteristic (ROC) curves for calculating the area under the curve (AUC values). For ssGSEA, the ‘GSVA’ package was utilized.

## 3 RESULTS

### 3.1 Methylation of RNA genes expressed differently between LUAD and paracancerous tissues

**FIGURE 1:**
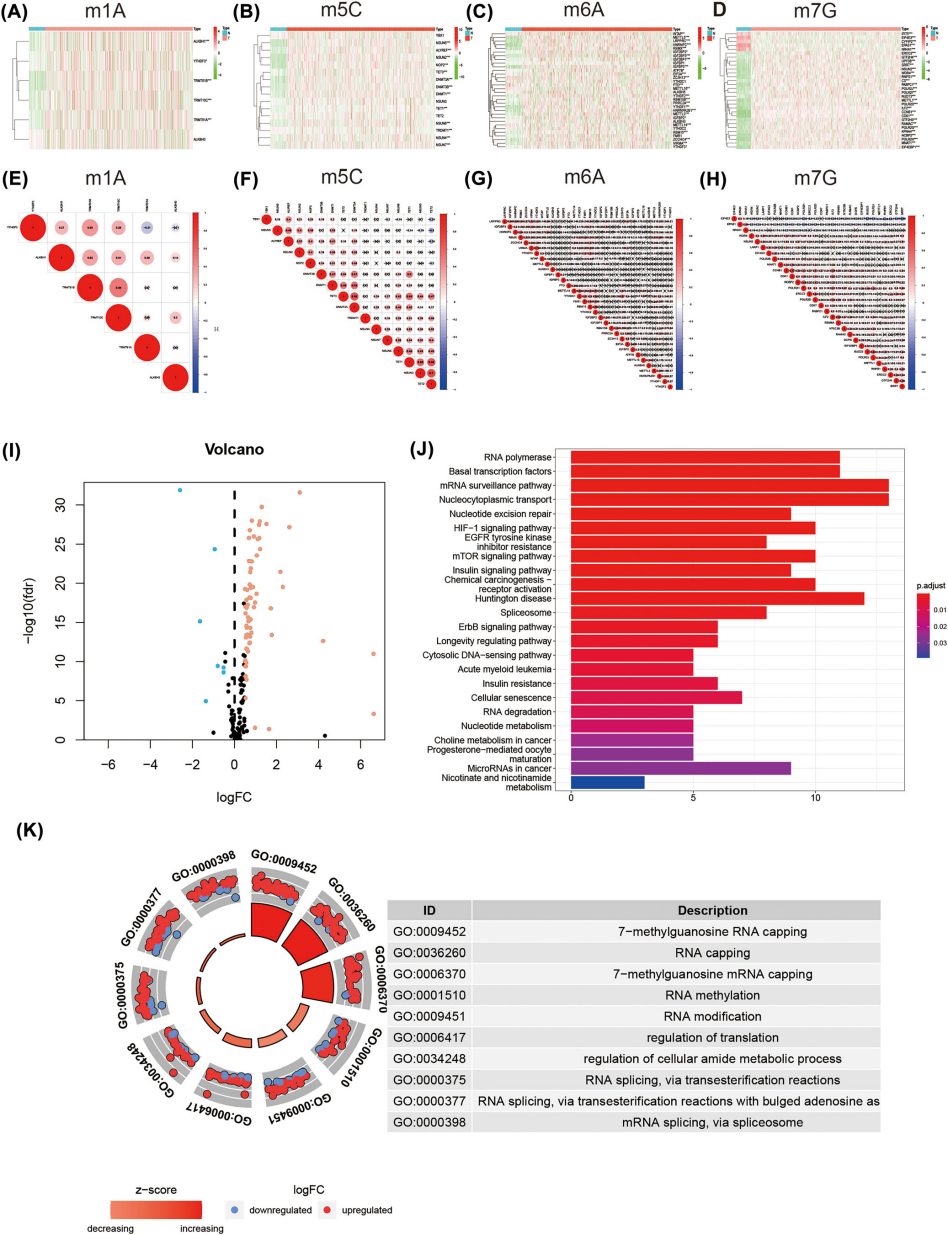
Differential expression of RNA methylation gene and enrichment analysis of DEGs associated with methylation in lung adenocarcinoma and paracancerous tissues. (A–D) Clustered heatmap of the expression patterns for DEGs in different RNA methylation modifications. (E–H) Correlation maps for DEGs associated different RNA methylation modifications. (I) Volcano plot. Seventy‐one DEGs related to RNA methylation are observed; 64 up‐regulated genes are coloured in red (red dots); seven down‐regulated genes are coloured in blue (blue dots). (J) KEGG pathway of DEGs associated with methylation. (K) GO enrichment analysis of DEGs associated with methylation. Abbreviations: DEGs, differentially expressed genes; FC, fold change; FDR, false discovery rate; GO, gene ontology; KEGG, Kyoto Encyclopedia of Genes and Genomes.

From TCGA‐LUAD, we downloaded 594 LUAD and paracancerous tissue data. We screening out 165RNA‐methylation‐related genes, including 6 m1C, 8 m5C, 35 m6A and 116 m7G. Subsequently, the different expression levels of the above four groups of RNA methylation‐related genes in cancer tissues and adjacent tissues were analysed, resulting in a total of 71 DEGs being screened out (Figure 1A–D). In addition, we examined the relationship between the methylation‐related RNA DEGs in each group (Figure 1E–H).

### 3.2 Enrichment analysis of methylation‐related lncRNA DEGs

Among the 71 methylation‐related RNA DEGs (DEGs), seven were down‐regulated and 64 were up‐regulated (Figure 1I). KEGG pathway analysis indicated the significant enrichment pathways associated with the 71 RNA methylation‐associated DEGs are RNA polymerase, basal transcription factors, mRNA surveillance pathway, nucleocytoplasmic transport, nucleotide excision repair, and so on (Figure 1J). GO analysis revealed that the function of DEGs was primarily enriched in the regulation of translation, RNA capping, RNA methylation, 7‐methylguanosine RNA capping, and so on (Figure 1K).

### 3.3 Methylation‐related lncRNA signatures predicting LUAD

The ‘LimmaR’ package was used to identify 1598 methylation‐related lncRNAs. In a univariate regression analysis, it was found that 83 of these variables were related to the prognosis of patients with LUAD. Multivariate Cox regression analysis was performed to screen out 18 methylation‐related lncRNAs (AP002761.1, AL118558.3, CH17‐340M24.3, AL353150.1, AC004687.1, LINC00996, AF186192.1, HSPC324, AC087752.3, FAM30A, AC106047.1, AC026355.1, ABALON, LINC01843, AL606489.1, NKILA, AP001453.2 and GSEC). We used these 18 lncRNAs independent predictors to build a prediction model. Figure 3A‐i illustrates the expression levels of 18 lncRNA signatures in the tissue of LUAD. Using the ‘gg alluvial’ R package and Cytoscape, we found 18 pairs of lncRNA‐mRNA co‐expressed in the network (Figure 3A‐ii, |*R* ^2^| > 0.4 and *P* < 0.001). Table 1 illustrates the co‐expression relationship. Based on this, we further visualized the corresponding Sankey diagram showing the relationship between prognostic lncRNAs and risk type (Figure 3A‐iii). In total methylation‐related lncRNAs, ABALON, AC004687.1, AC087752.3, AL606489.1, AP001453.2, CH17‐340M24.3, GSEC and NKILA were risk factors. AP002761.1, AL118558.3, AL353150.1, LINC00996, AF186192.1, HSPC324, FAM30A, AC106047.1, AC026355.1 and LINC01843 were identified as protective lncRNAs. The risk scores were analysed as follows: riskscore = (−0.730145377 × AC004687.1 expression) + (1.411577652 × AC087752.3 expression) + (0.763757779 × AC034236.2 expression) + (0.516345501 × NKILA expression) + (−0.922753388 × AL118558.3 expression) + (0.78048419 × ABALON expression) + (0.493697701 × CH17‐7340 M24.3 expression) + (−0.933963185 × FAM30 expression) + (−0.297718694 × LINC01843 expression) + (−0.924673401 × LINC00996 expression) + (0.751538891 × AP001453.2 expression) + (−0.457662074 × AL353150.1 expression) + (0.60638866 × AL606489.1 expression) + (−0.548720538 × AC026355.1 expression) + (−0.666023466 × AF186192.1 expression) + (−0.854079483 × AP002761.1 expression) + (−0.539050891 × HSPC324 expression) + (0.536675573 × GSEC expression).

**TABLE 1:** The clinical characteristics of patients in different cohorts.

Variable	Entire	Validation cohort	
	TCGA dataset (*n* = 477)	First cohort (*n* = 240)	Second cohort (*n* = 237)
Age(%)			
Age ≤ 65	230(48.2)	105(43.8)	125(52.7)
Age > 65	247(51.8)	135(56.2)	112(47.3)
Gender(%)			
Female	257(53.9)	138(57.5)	119(50.2)
Male	220(46.1)	102(42.5)	118(49.8)
Stage(%)			
I + II	366(76.7)	177(73.8)	189(79.7)
III + IV	111(23.3)	63(26.2)	48(20.3)
*T* (%)			
*T*1 + *T*2	413(86.6)	199(82.9)	214(90.3)
*T*3 + *T*4	61(12.8)	39(16.3)	22(9.3)
TX + Unknow	3(0.6)	2(0.8)	1(0.4)
*M* (%)			
*M*0	313(65.6)	161(67.8)	152(64.1)
*M*1	24(5.0)	13(5.4)	11(4.6)
MX + Unknow	140(29.4)	66(27.5)	74(31.2)
*N* (%)			
*N*0	307(64.4)	153(63.8)	154(65.0)
*N*1 + *N*2	158(33.1)	79(32.9)	79(33.3)
*N*3	2(0.4)	2(0.8)	0(0)
NX + Unknow	10(2.1)	6(2.5)	4(1.7)

*Note*: To verify the applicability of the predictive signature for OS based on the entire TCGA dataset, we randomly divided the 477 LUAD patients into two cohorts (*n*_1_ = 240, *n*_2_ = 237). The demographic characteristics of patients in the two cohorts are shown in this table.

Abbreviations: M, metastasis; N, lymph node; T, tumour.

### 3.4 Analyse the apparent regulation of 18 methylation‐related lncRNA and their roles in the process of tumourigenesis and development

**FIGURE 2:**
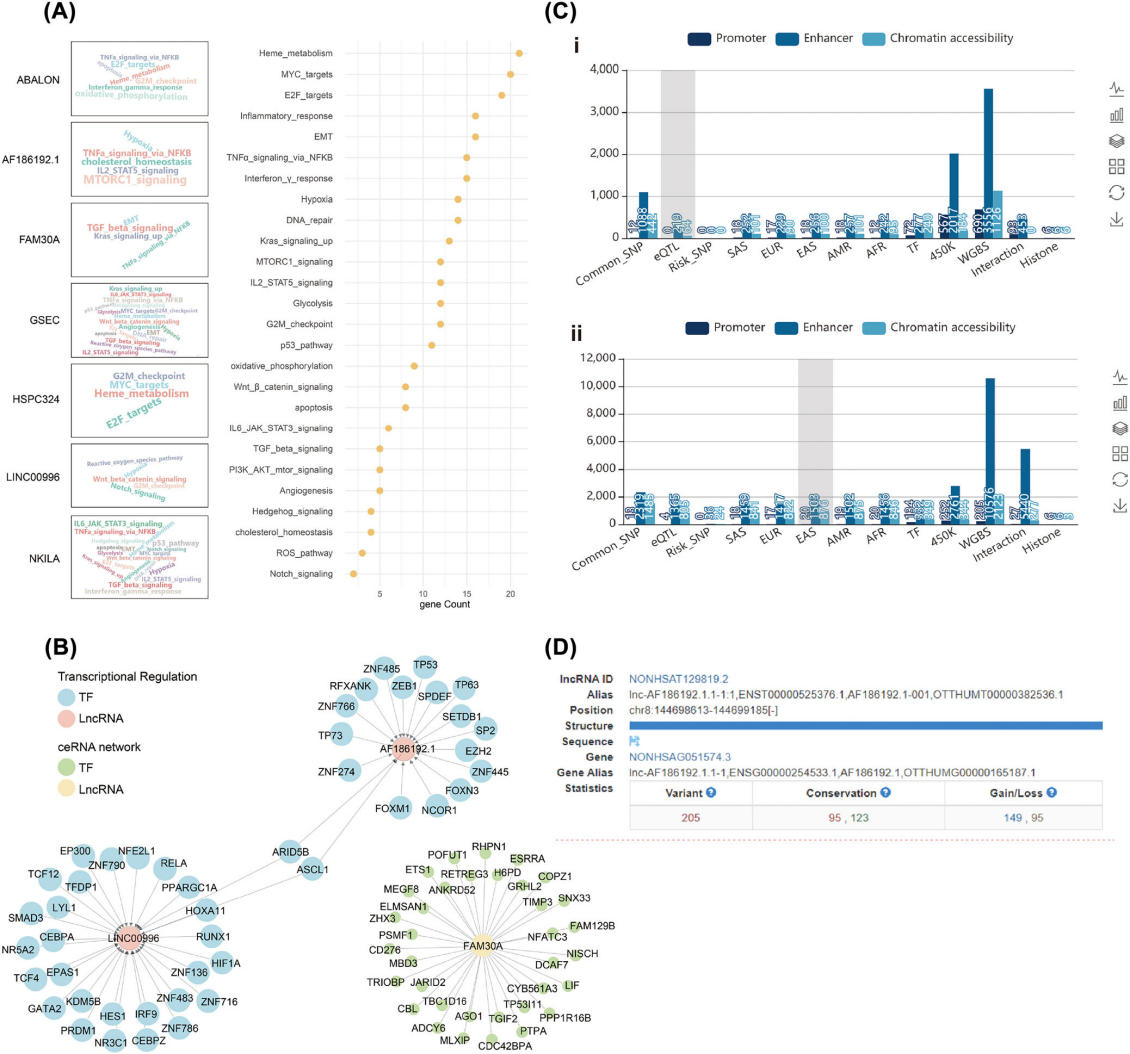
The apparent regulation of 18 methylation‐related lncRNA. (A) The enriched pathways associated with proteins that interact with 18 lncRNAs. (B) Transcription factors involved in the regulatory relationship with LINC00996 and AF186192.1, and the ceRNA network of LncRNA FAM30A. (C) The distribution of SNP loci in the enhancer region of AF186192.1 and LINC00996. (i) The SNP loci in the enhancer region of AF186192.1. (ii) The SNP loci in the enhancer region of LINC00996. (D) The SNP distribution, conservation, and miRNA:LncRNA binding interactions of LINC00996 and AF186192.1.

In order to analyse the role of these 18 lncRNAs in the process of tumourigenesis and development, we downloaded term set from LncSEA website and then used the clusterProfiler package of R language to analyse which term set in each file contains the 18 lncRNAs we need to identify and used the file ‘RNA_Protein_Interaction.gmt’ for enrichment analysis. It was found that the enriched pathways enriched by proteins interacting with 18 lncRNAs were mainly EMT, hypoxia, stemness and proliferation (Figure 2A), among which LINC00996 and AF186192.1 were regulated by multiple tumour‐related transcription factors, such as TP53 and TP63 (Figure 2B), and FAM30A and mRNA formed a ceRNA network (Figure 2C‐i,ii). Then TRlnc and LncRNASNP were used to analyse the SNP sites of lncRNAs, and 2319 SNP sites were identified in LILNC00996, 36 of which were risk SNP sites and 205 SNP sites in AF186192.1. Conservation indicates that AF186192.1 affects 95 conserved miRNAs and 123 non‐conserved miRNAs. Gain/loss indicates that af186192.1 promotes the binding of 149 pairs of miRNA:lncRNA and inhibits the binding of 95 pairs of miRNA:lncRNA (Figure 2D).

### 3.5 The predictive value of the lncRNAs signature towards LUAD prognosis

**FIGURE 3:**
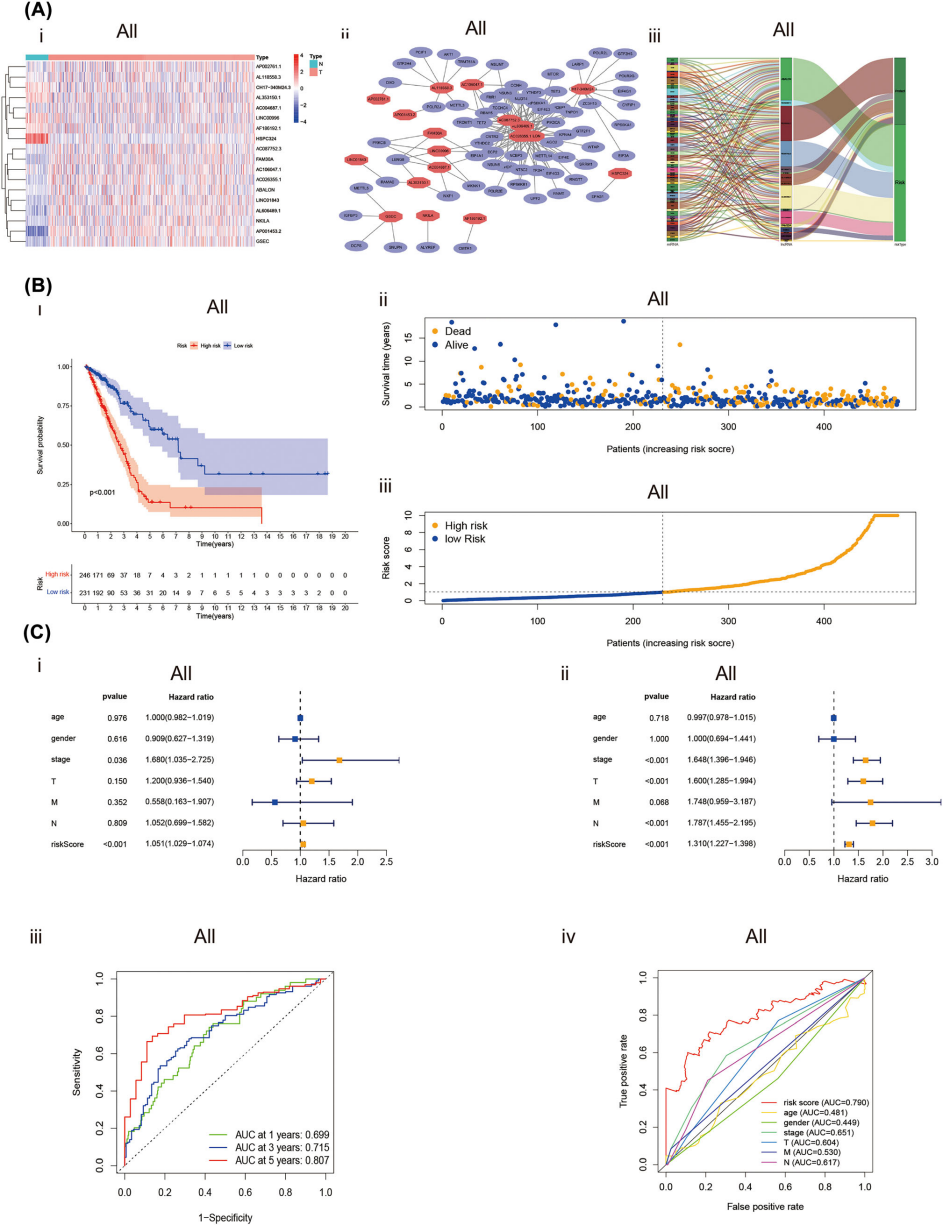
Expression and prognostic relevance of methylation‐related lncRNAs in LUAD. (A) Expression levels of total methylation‐related lncRNAs and their mRNA‐lncRNA networks. (i) Melitation‐related lncRNAs expressed differently in normal and LUAD tissues are depicted in the heatmap. (ii) Network of prognostic lncRNAs with co‐expressed methylation‐related DEGs in LUAD. (iii) Sankey coexpression diagram of methylation‐related lncRNAs signature. (B) The prognostic value of Predictive signatures for TCGA‐LUAD. (i) Kaplan‐Meier survival analysis curver of the risk score. (ii) Deaths and survivors of TCGA‐LUAD with different risk scores. Survivors are shown in blue, while deaths are shown in yellow. (iii) Risk scores distribution of TCGA‐LUAD. (C)Independent analysis of prognostic value of risk signature and clinicopathological variables. (i) Forest plot display the univariate Cox regression analysis. (ii) Forest plot display the multivariate Cox regression analysis. (iii) Time‐dependent ROC curves analysis for risk score's 1‐, 3‐, 5‐year survival outcome. (iv) ROC curve analysis of risk score and clinicopathological variables. Abbreviations: AUC, area under the curve; DEGs, differentially expressed genes; lncRNAs, long noncoding RNAs; LUAD, lung adenocarcinoma; M, metastasis; N, normal; N, lymph node; ROC, receiver operating characteristic; T, tumour.

**FIGURE 4:**
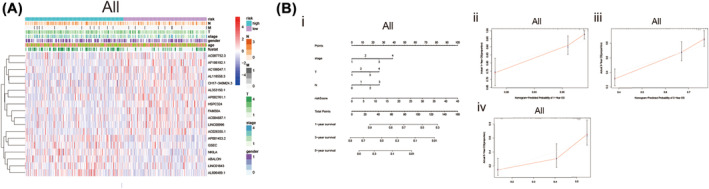
Nomogram and calibration curves for methylation‐based lncRNA prognostic signature. (A) The heatmap was used to depicte the relationship between clinicopathological variables and 18 fustat 1 prognostic lncRNAs based on methylation in the low‐ and high‐risk group. (B) Nomogram and the calibration curve of it. (i) A nomogram constructed with stage, T‐stage, N‐stage and the risk score based on predicted signature of lncRNAs analysis the 1‐, 3‐, 5 years overall survival for patients in LUAD. (ii–iv) Calibration curves constructed to compare the actual and predictive overall survival rates at 1, 3 and 5 years for verify the predictive value of nomogram. Abbreviations: lncRNAs, long noncoding RNAs; LUAD, lung adenocarcinoma; N, lymph node; OS, overall survival; T, tumour.

According to the cutoff value based on the median risk score we set, LUAD patients with different risk scores counted by the formula were assigned to high‐ and low‐risk groups. The Kaplan–Meier curve was used to estimate the overall survival (OS) time of LUAD patients in different risk score groups. As excepted, the OS time of the high‐risk group was shorter than the low‐risk group (Figure 3B‐i, *P* < 0.001). As shown in the curve of risk score and scatter plot, LUAD patients with significantly lower survival time had an increased risk score (Figure 3B‐ii,iii). Through the univariate and multivariate COX regression analysis. The lncRNA signature‐based risk score was verified the same independent prognostic value as some common clinicopathological parameters (Figure 3C‐i,ii). The AUC value for risk score, age, gender, stage, T stage, N stage and M stage were 0.790, 0.481, 0.449, 0.651, 0.604, 0.530 and 0.617, respectively (Figure 3C‐iii), which indicated the prognostic value of risk score in the prognosis of LUAD patients was superior to clinicopathological variables. This signature has well predictive value within 3 years. The AUCs value of risk score at 1, 3 and 5 years were 0.699, 0.715 and 0.807, respectively (Figure 3C‐iv). A heat map was constructed to analyse the expression level of 18 methylation‐related lncRNAs and the distribution of clinicopathological variables in different risk groups (Figure 4A). We also plotted a nomogram, incorporating clinicopathological variables and risk scores, which was used to predict the prognosis of patients with LUAD at 1, 3 and 5 years (Figure 4B‐i, *P* = 0.05). According to the calibration curve for the nomogram (Figure 4B‐ii, iii, iv), we further confirmed the predictive value of the nomogram for the prognosis of patients with LUAD.

### 3.6 Evaluate the independent predictive ability of lncRNA‐signature in different subgroups based on clinicopathological factor

**FIGURE 5:**
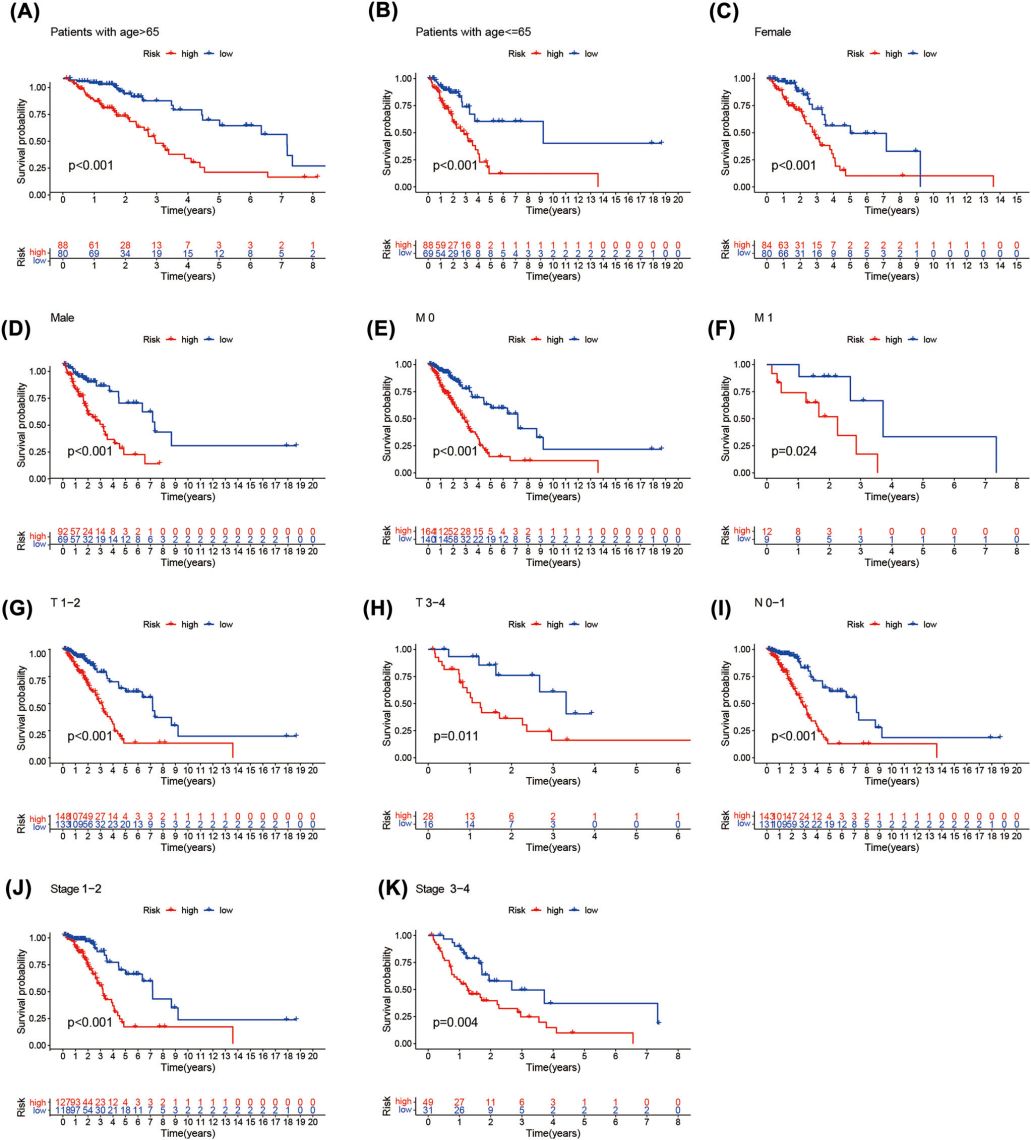
Kaplan–Meier survival curves of lung adenocarcinoma patients in low‐risk group and high‐risk group accroding to different clinicopathological variable groupss. (A, B) Age. (C, D) Gender. (E, F) M Stage. (G, H) T stage. (I) N stage. (J, K) stage. Abbreviation: N, lymph node; T, tumour.

To examine whether the predictive signature has an independent prognostic value for the patients in LUAD, first, we grouped LUAD patients according to different clinicopathological factors, and then performed Kaplan–Meier analyses to plot the survival curve for LUAD patients in different risk groups. Kaplan–Meier results show the OS rate of the patients in low‐risk is higher than the patients in high‐risk groups (Figure 5A–K). Predictive signatures can predict the prognosis of LUAD patients independently regardless of clinicopathological variables.

### 3.7 Validation of the prognostic value of lncRNA‐signature

**FIGURE 6:**
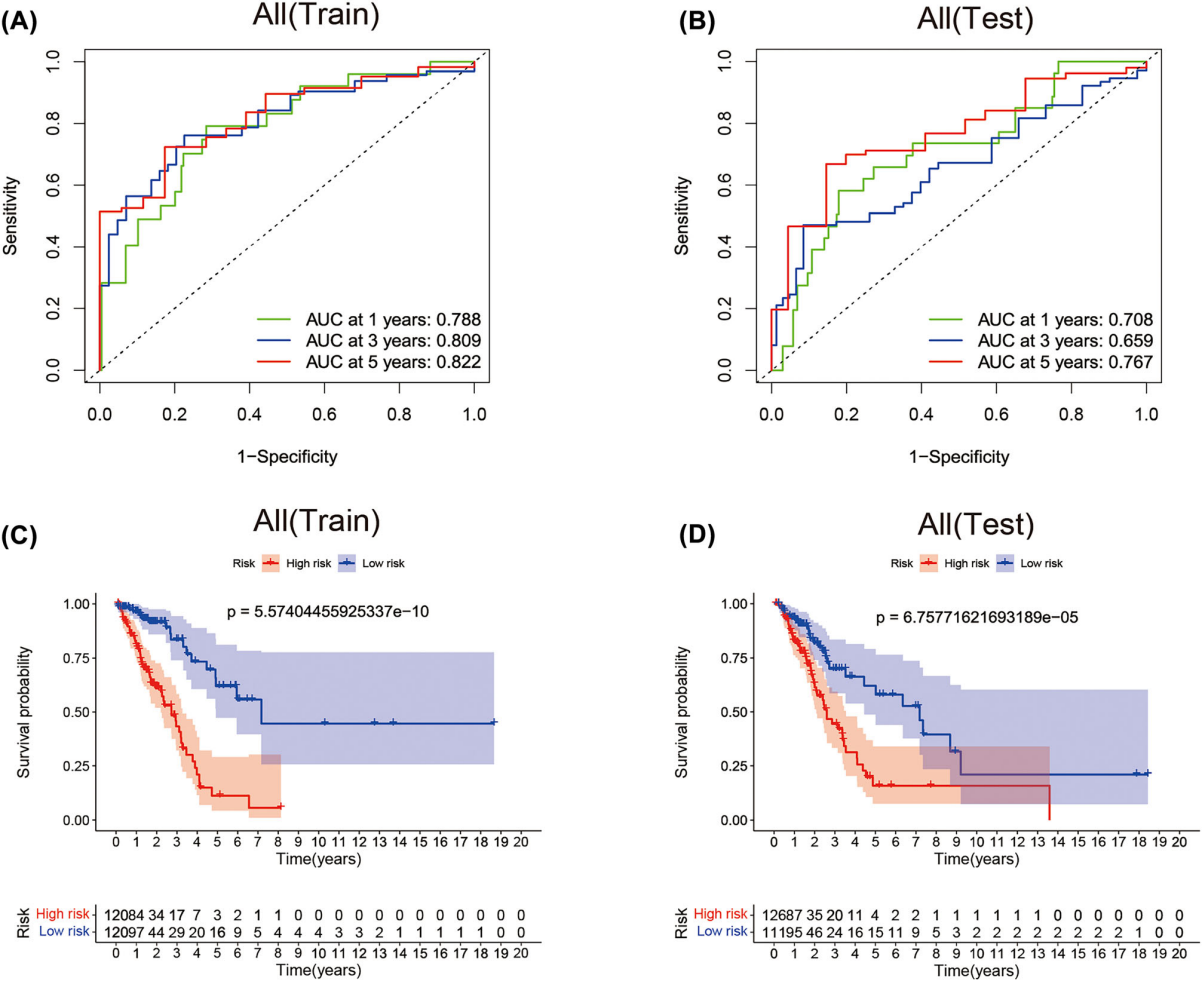
Internal validation for prognostic significance of the lncRNA signature. (A) ROC curve for validating the prognostic value of the lncRNA signature at 1‐, 3‐, 5‐year in training cohort. (B) ROC curve for validating the prognostic value of the lncRNA signature at 1‐, 3‐, 5‐year in test cohort. (C) Kaplan–Meier analysis for the training cohort show the worse OS time of lung adenocarcinoma (LUAD) patient in high‐risk group. (D) Kaplan–Meier analysis for the test cohort show the worse OS time of LUAD patient in high‐risk group. Abbreviations: AUC, area under the curve; OS, overall survival; ROC, receiver operating characteristic.

All patients (477)with LUAD acquired from the TCGA database were sorted into two cohorts, namely, the training group and the Test group (*n*_1_ = 240, *n*_2_ = 237), to test the applicability of the predictive signature. In Table 1, we present the demographic characteristics of both groups. As we excepted, The AUC of the 1‐, 3‐ and 5‐year ROC curves of the training group were respectively 0.788, 0.809 and 0.822(Figure 6A). Accordingly, test group survival rates of 1‐, 3‐ and 5 years were 0.708, 0.659 and 0.767 (Figure 6B). Both cohorts showed good predictive performance on ROC curves. The overall survival rate of the high‐risk group is higher than the low‐risk group both in the training group (Figure 6C, *P* = 5.574e−10) and the test group (Figure 6D, *P* = 67577e−5).

### 3.8 GSEA enrichment analysis

**FIGURE 7:**
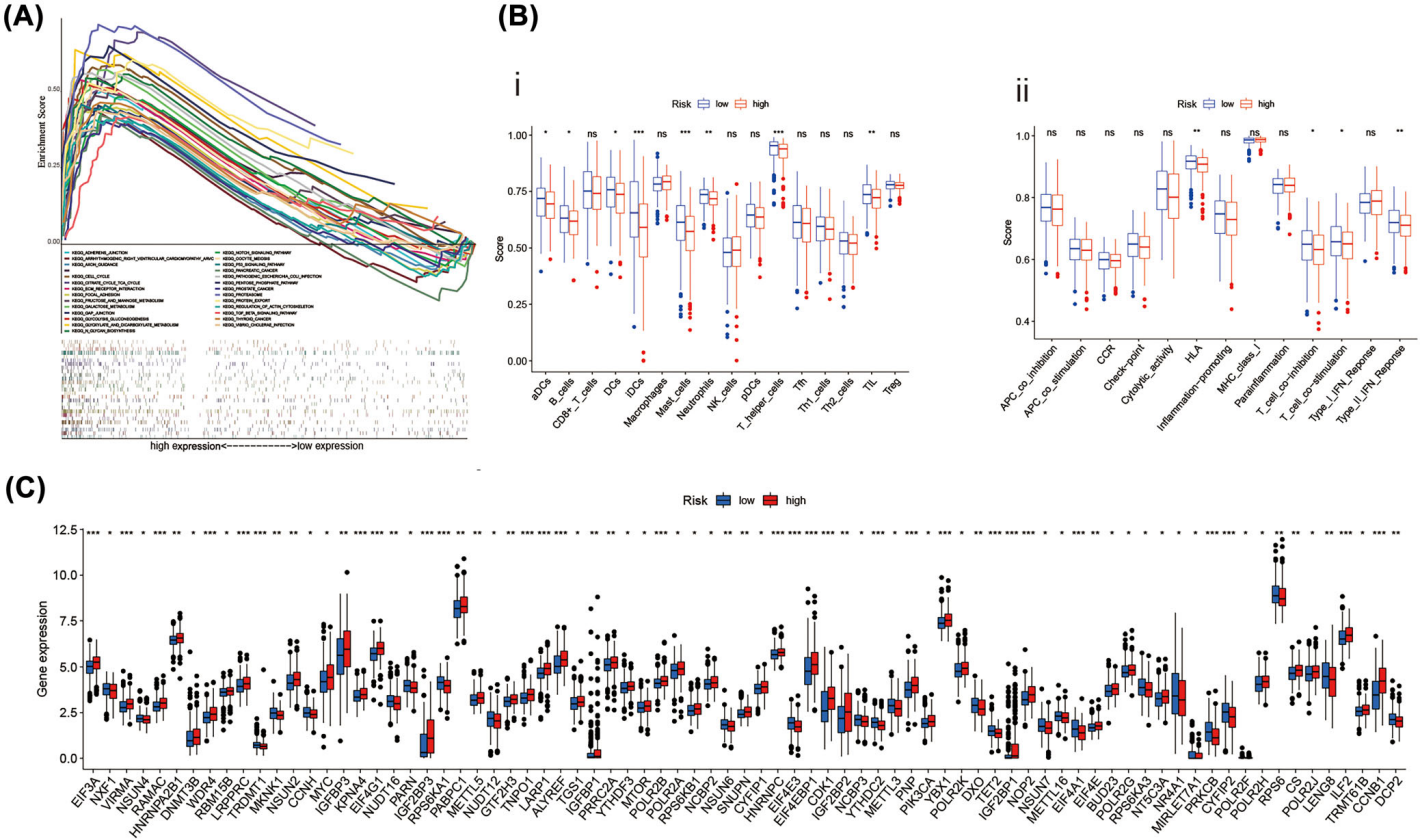
Immune profile differences in lung adenocarcinoma (LUAD) by lncRNA expression risk groups. (A) Gene set enrichment analysis of pathway. (B) A comparison of high‐ and low‐risk groups in terms of immune infiltrating cells and immune‐related functions using ssGSEA algorithm. (i) The infiltration levels of immune cells in two risk groups. (ii) The expression levels of immune functions in two risk groups. (C) Genes expression level of immune checkpoint between high and low risk groups. Abbeviations: KEGG, Kyoto Encyclopedia of Genes and Genomes; ssGSEA, single sample gene set enrichment analysis. *P < 0.05; **P < 0.01; ***P < 0.001; ns, non‐significant.

GSEA was performed to analyse the different expression levels of tumour‐ and immune‐related pathways in the high‐ and low‐risk groups. Results show that tumourigenesis and immune‐related pathways, such as pentose‐phosphate‐pathway, cell cycle pathway, N‐glycan‐biosynthesis, glycolysis‐gluconeogenesis, p53‐signalling‐pathway, notch signalling pathway, TGF‐beta‐signalling‐pathway, pancreatic cancer, thyroid cancer and prostate cancer were significantly enriched in the high‐risk group (Figure 7A).

### 3.9 The immune landscape of LUAD patients in different risk groups

We also performed ssGSEA for revealing the immune landscape of the LUAD patients with low or high‐risk scores. The infiltration difference of 16 immune cells was analysed, and the results showed in Figure 7B‐i that eight immune cell infiltration differences between the high‐risk group and the low‐risk group. Among these eight immune cells, in the low‐risk group, immature dendritic cells (iDCs), mast cells and T helper cells were significantly infiltrated more than in the high‐risk group. Next, the expression level of representative immune signalling pathways in LUAD patients within different risk groups was evaluated. The results show that the immune function score of human leukocyte antigen (HLA), type IIIFN response was significantly higher in the low‐risk group (Figure 7B‐ii). These results indicate that Immune function activation seems to be more prone for High‐risk individuals. Cancer is associated with immune checkpoint genes. Based on our analysis, some immune checkpoint genes were expressed differently in the two risk groups (Figure 7C).

### 3.10 Evaluating the prognostic predictive effect of prognostic signals by principal component analysis

**FIGURE 8:**
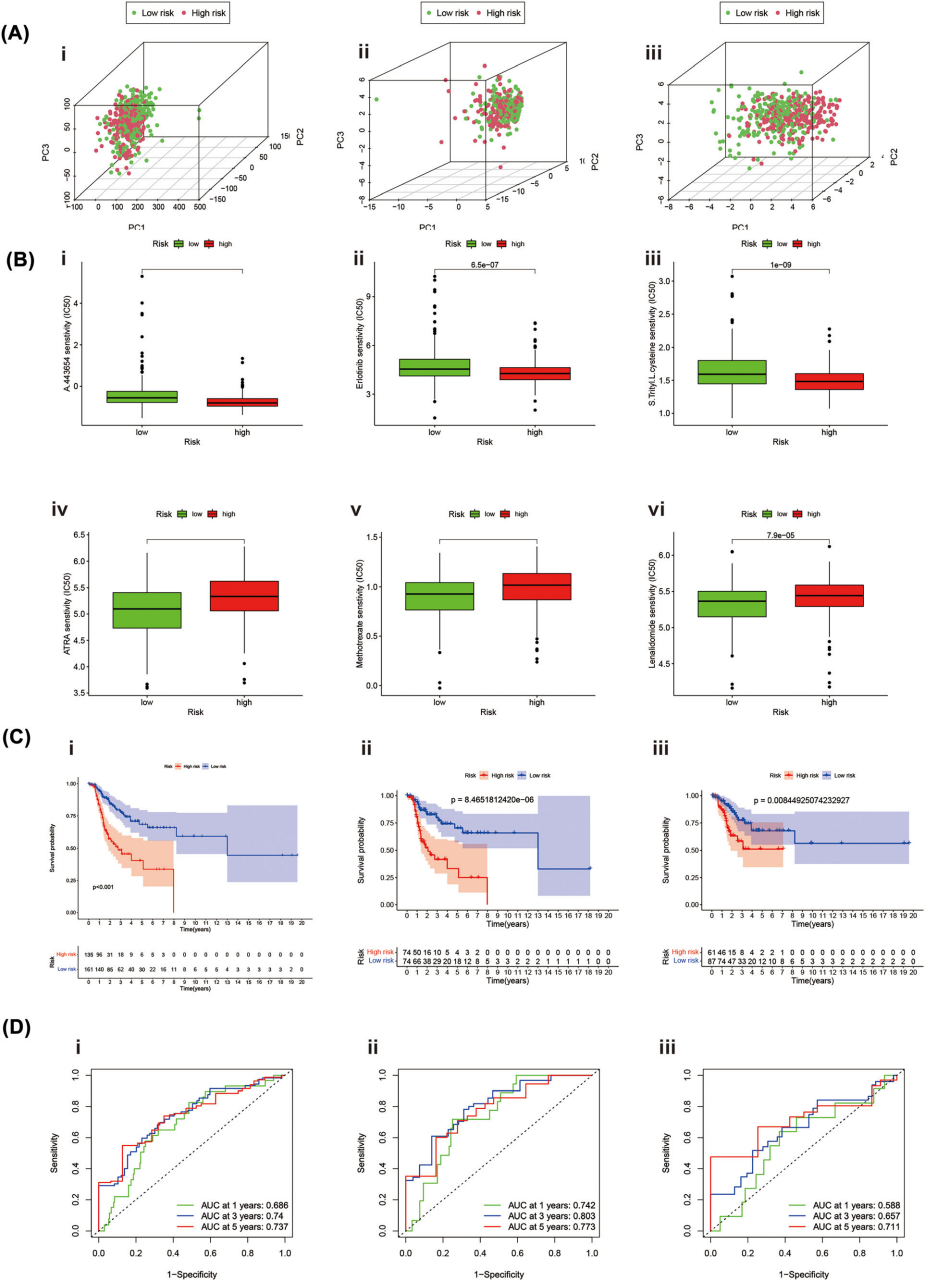
Principal component analysis (PCA) of methylation profiles and survival analysis stratified by lncRNA risk in lung adenocarcinoma (LUAD). (A) PCA shows the distribution of TCGA‐LUAD. (i) PCA analysis of total methylation‐related genes. (ii) PCA analysis of total methylation‐related lncRNAs. (iii) PCA analysis of lncRNAs‐signature. (B) Drug sensitivities between two risk groups. (i–iii) The high‐risk group IC50s for A.443654, ErIotinib and S.Trityl.L. Steine was higher than the low‐risk group. (iv–vi) The low‐risk patients IC50s for ATRA, methotrexate and lenalidomide were higher than the high‐risk group. (C, D) The predictive efficacy of the methylation‐lncRNA signature for DFS. (C‐i) Kaplan–Meier survival curve for overall dataset. (C‐ii) Kaplan–Meier survival curve for the first cohort. (C‐iii) Kaplan–Meier survival curve for the second cohort. (D‐i) ROC curves in the entire dataset. (D‐ii) ROC curve in the first cohort. (D‐iii) ROC curve the second cohort. Abbreviations: AUC, area under the curve; DFS, disease‐free survival; lncRNAs, long noncoding RNAs; ROC, receiver operating characteristic.

We performed principal component analysis to visualize the spatial distribution of LUAD patients in high‐ and low‐risk. We used PCA profiles to depict patients based on total methylation‐related genes, total methylation‐related lncRNA, and lncRNA signature. According to the prediction signal constructed using lncRNA‐signature, the lowest and highest risk groups showed the most evident differences (Figure 8A‐i, ii, iii). This indicated that our prediction signal based on total methylation‐related lncRNA could distinguish well between high‐risk and low‐risk groups.

### 3.11 Correlation of the predicted signals with LUAD treatment

An analysis of the correlation between the predicted signals and conventional LUAD chemotherapy drugs was performed. The high‐risk group IC50s for A.443654, ErIotinib, and S.Trityl.L. Steine was higher than the low‐risk group (Figures 8B‐i, ii, iii). Nevertheless, low‐risk patients received less ATRA, Methotrexate, and Lenalidomide (Figures 8B‐iv, v, vi). Data from these studies can be used to reveal individualized treatment options for LUAD patients.

### 3.12 A methylation‐related long noncoding RNA predictive signature for DFS is constructed

Disease‐free survival (DFS) is useful when screening LUAD patients. Also, methylation‐related lncRNAs for total lncRNAs were developed as a predictor of DFS. From the cBioPortal database, we downloaded DFS data for 567 patients with LUAD. Based on univariate analysis, we identified 22 RNA methylation‐related lncRNAs that were considerably associated with DFS. As a result of multivariate COX analysis, we obtained four lncRNAs associated with RNA methylation and used these four lncRNAs to construct predictive features. Calculate the risk score as follows:riskscore = (1.127506988 × AC127502.2 expression) + (0.595996496 × LINC00511 expression) + (−0.661724435 × AL139289.2expression) + (0.55340284 × UBE2Q1‐AS1 expression). LUAD patients with different risk scores, calculated with the formula, were divided into high‐ and low‐risk groups based on the median risk. Kaplan–Meier survival curve analysis shows the results that DFS in a high‐risk group is shorter (Figure 8C‐i). The AUC of 1‐, 3‐ and 5‐year survival were 0.686, 0.74 and 0.734 (Figure 8D‐i). Two internal cohorts(*n*_1_ = 148; *n*_2_ = 150) of 298 LUAD patients were randomly assigned to verify the predictability of DFS. The DFS and AUC trends results, calculated with the Kaplan–Meier, in the firster internal cohorts (Figure 8B‐ii, iv) or second internal cohort (Figure 8D‐iii, vi) were consistent with the whole LUAD patients dataset.

## 4 DISCUSSION

More and more evidence shows that abnormal epigenetic modification of RNA leads to the occurrence of cancer to a certain extent, among which RNA methylation modification is a hot topic in the field of cancer research in recent years.^6^, ^7^ It has been reported that N6‐methyladenosine (m6A), as one of the most common RNA methylation modifications, is involved in the occurrence and development of many diseases and tumours. 98% of the known RNA sequences cannot be translated into peptides. Among them, long non‐coding RNAs (lncRNA) with a length of more than 200 nt have multiple interaction sites, so they are responsible for a large number of biological functions.^8^ Studies have shown that the abnormal abundance of methylation‐modified lncRNAs in tumour tissues is often related to the prognosis and treatment response of cancer patients.^9^ For example, overexpression of NEAT1, one of the m6A‐modified lncRNA, often leads to bone metastasis of cancer cells, and it significantly increases the risk of bone metastasis of prostate cancer through RNA–DNA interaction.^8^ In breast cancer cells, the abundance of lncRNA modified by ALYREF is high, which promotes the development of tumours by affecting the apoptosis and mitochondrial energy metabolism of cancer cells.^8^ In Hepatocellular Carcinoma (HCC), nine M7G‐associated lncRNA were identified as reliable prognostic signatures for HCC.^10^

Adenocarcinoma of the lung is one of the most common malignant tumours in the lung. This disease is characterized by poor prognosis and high mortality, which is a great threat to the health of people all over the world.^11^ Efficient and accurate biomarkers are of great significance for improving the diagnosis and treatment of LUAD patients. At present, the research on methylation‐related lncRNAs in LUAD is still in the preliminary stage.^12^, ^13^ Given the prognostic power of lncRNAs associated with methylation in other cancers, a comprehensive prognostic risk model of lncRNAs associated with methylation was constructed and evaluated about the relevant clinical characteristics of LUAD patients to validate its potential as a novel biomarker for LUAD diagnosis and prognosis as well as immunotherapy response.^11^, ^14^

In this study, based on the public TCGA database, we first screened 71 differentially expressed methylated genes in LUAD patients. According to KEGG enrichment analysis, the DEGs are mainly enriched in the RNA polymerase pathway, basal transcription factors pathway, mRNA surveillance pathway, nucleocytoplasmic transport pathway and nucleotide excision repair pathway. Research has shown that targeting mutant dicer tumourigenesis in pleuropulmonary blastoma via inhibition of RNA polymerase I.^15^ Regulation of basal transcription factors in hepatocellular carcinoma promotes the occurrence and development of hepatocellular carcinoma.^16^ The over‐activation of The NMD mRNA surveillance pathway is one of the causes of the deterioration of hereditary diffuse gastric cancer (HDGC).^17^ Various nucleotide excision repair (NER) gene alteration sites have become potential cancer therapeutic targets.^18^ These results suggest that methylation‐related genes may regulate the development of LUAD through the above‐mentioned pathways. However, the specific mechanism of these pathways in LUAD remains to be further studied.

Univariate Cox regression analysis and multivariate Cox regression analysis were used to constructing methylation‐related lncRNA signatures to predict the prognosis of LUAD patients. The methylation related lncRNAs signature consists of 18 lncRNAs:AP002761.1, AL118558.3, CH17‐340 M24.3, AL353150.1, AC004687.1, LINC00996, AF186192.1, HSPC324, AC087752.3, FAM30A, AC106047.1, AC026355.1, ABALON, LINC01843, AL606489.1, NKILA, AP001453.2, GSEC. We also found mRNA (ABALON, AC004687.1, AC026355.1, AC087752.3, AC106047.1, AF186192.1, AL118558.3, AL353150.1, AL606489.1, AP001453.2, AP002761.1, CH17‐340 M24.3, FAM30A, GSEC, HSPC324, LINC00996, LINC01843 and NKILA) significantly co‐expressed with these lncRNAs. According to the cutoff value based on the median risk score we set, LUAD patients with different risk scores calculated by the formula were assigned to the high‐ and low‐risk group. As excepted, the OS time of the high‐risk group was shorter than the low‐risk group. A multivariate and univariate Cox regression analysis revealed that risk score‐based lncRNAs signature verified the same independent prognostic value. The ROC curve indicated the prognostic value of risk score in the prognosis of LUAD patients was superior to common clinicopathological variables. Internal validation confirmed the good predictive performance of the predictive signature.

We used two websites TRLnc and LncRNASNP to analyse the SNP sites of lncRNAs, and a total of two of 18 lncRNAs were retrieved from TRlnc: AF186192.1 and LINC00996. Among the 18 lncRNAs, lncRNASNP only retrieved: AF186192.1. At the same time, through the analysis of these 18 lncRNAs, we found that the enriched pathways of proteins that bind to 18 lncRNAs are mainly EMT, hypoxia, stemness, proliferation and so on, among which LINC00996 and AF186192.1 are regulated by multiple tumour related transcription factors, such as TP53 and TP63, and fam30a and mRNA form a Cerna network. At the same time, 2319 SNP loci were found in LINC00996, 36 of which were risk SNP loci, and 205 SNP loci were found in AF186192.1: it means that AF186192.1 affects 95 conserved miRNAs and 123 non conserved miRNAs; AF186192.1 promotes the binding of 149 pairs of miRNA:LncRNA and inhibits the binding of 95 pairs of miRNA:lncRNA.

According to the results of GSEA, we know that Pentose‐phosphate‐pathway, Cell cycle pathway, N‐glycan‐biosynthesis, Glycolysis‐gluconeogenesis, p53‐signalling‐pathway, Notch signalling pathway, and TGF‐beta‐signalling‐pathway mainly enriched in the high‐risk group. In previous studies, these tumourigenesis and immune‐related pathways were strongly associated with the prognosis of LUAD.^19^, ^20^, ^21^ Based on ssGSEA analysis, we found that in vivo in patients with LUAD, immature dendritic cells (iDCs), mast cells, and T helper cells were significantly infiltrated in the low‐risk group. Dendritic cells are important immune cells, and immature dendritic cells (iDCs) are the key targets to stimulate tumour immunity.^22^ Studies have shown that patients with extensive infiltration of mast cells in non‐small cell lung cancer (NSCLC) have prolonged survival. Similarly, in prostate cancer, researchers have found that intratumoural MCs infiltration may prevent prostate cancer recurrence.^23^, ^24^ CD4 + T helper cells also participate in anti‐tumour immunity through a variety of mechanisms.^25^ In addition to that, the immune function score of human leukocyte antigen (HLA), and type IIIFN response were significantly higher in the low‐risk group. In conclusion, the prognosis of patients in the low‐risk group is better than that in the high‐risk group, which may be related to their good tumour immune function. Most of the immune targets were significantly up‐regulated in the high‐risk group of patients tested, which supports the potential link of lncRNA risk score with immunotherapy and further predicts prognosis. Our research also shows that high‐risk patients are probably sensitive to A.443654, ErIotinib and S.Trityl.L. Steine. Principal component analysis verified that our prediction signal based on total methylation‐related lncRNAs could distinguish well between high‐risk and low‐risk groups.

However, there are some limitations to our study. First, we mainly use TCGA database internal validation and lacked external validation to assess the applicability of predictive signatures. Second, the mechanism of methylation‐related lncRNA in LUAD needs to be verified by clinical experiments.

## 5 CONCLUSIONS

Therefore, the lncRNA signature associated with methylation can independently predict LUAD prognosis. And we provide basic results for exploring the possible mechanism of methylation‐related lncRNA in the progression of LUAD, which can provide new possibilities for clinical treatment in LUAD. However, further experimental verification is still needed.

## AUTHOR CONTRIBUTIONS

All authors contributed to the study conception and design. The first draught of the manuscript was written by KunYang and HaoLiu. All authors commented on previous versions of the manuscript. All authors read and approved the final manuscript.

## CONFLICT OF INTEREST STATEMENT

The authors have no relevant financial or non‐financial interests to disclose.

## ETHICS STATEMENT

TCGA and GEO belong to public databases. The patients involved in the database have obtained ethical approval. Users can download relevant data for free for research and publish relevant articles. Our study is based on open source data, so there are no ethical issues and other conflicts of interest.

## Data Availability

These data were derived from the following resources available in the public domain: TCGA at https://portal.gdc.cancer.gov/; LncSEA at http://bioinformatics.ustc.edu.cn/lncsea/.
